# Biocompatible Electrochemical Sensor Based on Platinum-Nickel Alloy Nanoparticles for In Situ Monitoring of Hydrogen Sulfide in Breast Cancer Cells

**DOI:** 10.3390/nano12020258

**Published:** 2022-01-14

**Authors:** Asit Kumar Panda, Murugan Keerthi, Rajalakshmi Sakthivel, Udesh Dhawan, Xinke Liu, Ren-Jei Chung

**Affiliations:** 1 Department of Chemical Engineering and Biotechnology, National Taipei University of Technology (Taipei Tech), Taipei 10608, Taiwan; asitpanda6@gmail.com (A.K.P.); keerthimurugan1992@gmail.com (M.K.); rajalakshmicnr@gmail.com (R.S.); 2 Centre for the Cellular Microenvironment, University of Glasgow, Glasgow G12 8QQ, UK; Udesh.dhawan@glasgow.ac.uk; 3 College of Materials Science and Engineering, Shenzhen University, Shenzhen 518060, China

**Keywords:** hydrogen sulfide, endogenous gasotransmitter, PtNi alloy, live cells, electrochemical sensor

## Abstract

Hydrogen sulfide (H_2_S), an endogenous gasotransmitter, is produced in mammalian systems and is closely associated with pathological and physiological functions. Nevertheless, the complete conversion of H_2_S is still unpredictable owing to the limited number of sensors for accurate and quantitative detection of H_2_S in biological samples. In this study, we constructed a disposable electrochemical sensor based on PtNi alloy nanoparticles (PtNi NPs) for sensitive and specific in situ monitoring of H_2_S released by human breast cancer cells. PtNi alloy NPs with an average size of 5.6 nm were prepared by a simple hydrothermal approach. The conversion of different forms of sulfides (e.g., H_2_S, HS^−^, and S^2−^) under various physiological conditions hindered the direct detection of H_2_S in live cells. PtNi NPs catalyze the electrochemical oxidation of H_2_S in a neutral phosphate buffer (PB, pH 7.0). The PtNi-based sensing platform demonstrated a linear detection range of 0.013–1031 µM and the limit of detection was 0.004 µM (S/N = 3). Moreover, the PtNi sensor exhibited a sensitivity of 0.323 μA μM^−1^ cm^−2^. In addition, the stability, repeatability, reproducibility, and anti-interference ability of the PtNi sensor exhibited satisfactory results. The PtNi sensor was able to successfully quantify H_2_S in pond water, urine, and saliva samples. Finally, the biocompatible PtNi electrode was effectively employed for the real-time quantification of H_2_S released from breast cancer cells and mouse fibroblasts.

## 1. Introduction

Hydrogen sulfide (H_2_S) is the third most important endogenous gasotransmitter, followed by carbon monoxide (CO) and nitric oxide (NO) [1]. It is produced at a very low concentration in mammalian systems, mainly via a cysteine biosynthesis pathway by several enzymatic reactions to control several pathological activities such as cell proliferation, neurotransmission, antioxidation, and vascular inflammation [2]. H_2_S also plays other essential biological roles, including anti-inflammation, angiogenesis, inhibition of insulin signaling, and regulation of blood pressure [3]. However, the accumulation of H_2_S in the cellular environment is highly harmful to living organisms and can be related to several persistent diseases, such as Alzheimer’s disease, fatal kidney disease, diabetes, Parkinson’s disease, Down Syndrome, and even cancer [4]. Moreover, concentration imbalance of H_2_S in living organisms damages the neurotransmission, immune response, blood pressure regulation, endocrine, gastrointestinal, and circulatory systems [5,6]. Due to its involvement in a large number of physiological processes, it is essential to develop fast, sensitive, and convenient methods to monitor endogenous H_2_S in biological systems [7]. To date, various analytical methods for H_2_S detection have been developed, including fluorimetry, spectrophotometry, chromatography, and chemiluminescence [8,9,10]. Chemiluminescence spectroscopy is often limited to use in biological samples because of its high temperature, long recovery time, and slow response [11]. Fluorescence, instead of imaging H_2_S in live cells, is still unable to monitor real-time in situ fluctuations of H_2_S [12]. Electrochemical biosensors are more convenient for in situ real-time monitoring of H_2_S because of their high sensitivity, strong selectivity, rapidity, excellent reproducibility, ease of operation, and label-free detection [13,14]. However, the detection limit of H_2_S sensors still needs to be improved owing to the low concentration of H_2_S in the cells.

Recently, alloy NPs have gained considerable attention for various electrochemical applications. The noble metal platinum (Pt) is a commonly used electrocatalyst because of its superior catalytic activity [15,16]. Nevertheless, the catalytic performance of Pt nanoparticles can be hindered due to their tendency to self-aggregate [17]. Therefore, the alloying method has become a smart strategy for improving the electrochemical performance of Pt-based electrocatalysts, in which another metal is combined with Pt to form a Pt-M alloy (M = Ag, Au, Pd, Ru, and Ni), thus effectively modifying the electronic structure of Pt and its active surface sites [18,19]. Nickel (Ni) has been extensively applied in batteries, electroplating, and other applications owing to its high thermal resistance and excellent corrosion resistance. Nickel is also inexpensive and has high catalytic activity. PtNi bimetallic alloy NPs exhibit promising catalytic activity compared to pure Pt [19,20]. Alloying of Pt with Ni not only improves the catalytic activity but also decreases the cost of the material. Moreover, with the incorporation of Ni, the lattice of Pt shrinks, and the distance between adjacent Pt-Pt decreases. The decrease in the distance between adjacent Pt atoms facilitates the reaction rate, specific activity and thus improves the electrochemical performance.

**Scheme 1:**
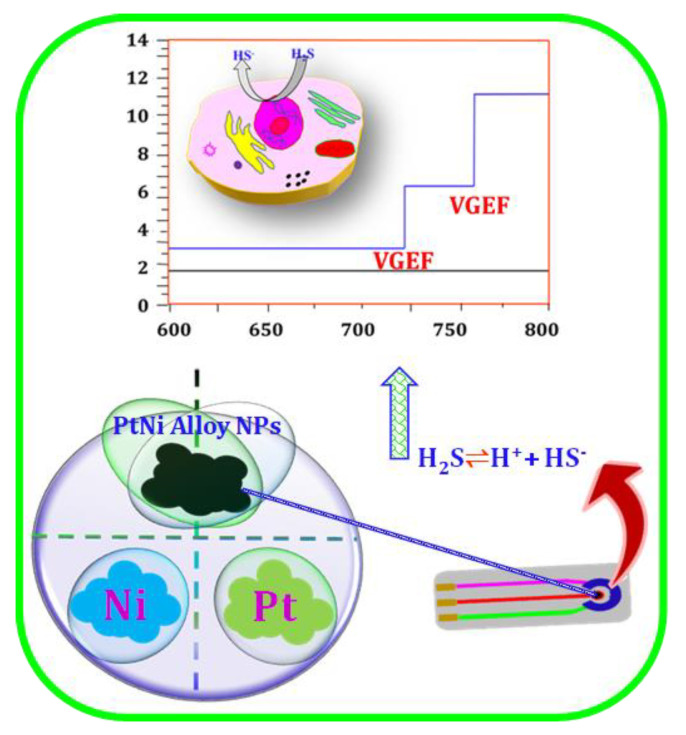
Schematic representation of PtNi alloy NP formation and electrochemical detection of H_2_S in a human breast cancer cell.

In this study, as shown in Scheme 1, PtNi alloy NPs were prepared by a hydrothermal method, and a modified PtNi electrode was employed for the detection of H_2_S. The crystalline structure, morphology, and elemental composition of the PtNi alloy NPs were determined using various characterization techniques. The electrocatalytic performance of the PtNi-modified electrode for the electrochemical detection of H_2_S was investigated using voltammetry. PtNi alloy NPs have higher specific surface area thereby increasing the catalytic active sites and stability of the SPE for the efficient detection of H_2_S. Moreover, the sensitivity and selectivity of the PtNi-modified electrodes were studied using an amperometric (i-t) method. Finally, the PtNi sensor was employed for the practical analysis of H_2_S in real samples, such as pond water, urine, and saliva samples.

## 2. Materials and Methods

### 2.1. Chemicals and Reagents

Platinum(II) acetylacetonate (Pt(acac)_2_, ≥97%), nickel(II) nitrate hexahydrate (Ni(NO_3_)_2_.6H_2_O, ≥99.9), ascorbic acid (AA, C_6_H_8_O_6_, ≥ 99.1%), polyvinylpyrrolidone (PVP, (C_6_H_9_NO)_n_), sodium sulfide (Na_2_S, ≥99.0%), and all other chemicals were purchased from Sigma-Aldrich (St. Louis, MO, USA). Sodium phosphate monobasic (NaH_2_PO_4_) and sodium phosphate dibasic (Na_2_HPO_4_) were used for the preparation of 0.05 M phosphate buffer (PB). Hydrochloric acid (HCl, 36.5–38.0%) and sodium hydroxide (NaOH, ≥98.0%) were used to adjust the pH of the PB. Double distilled (DD) water and ethanol were used for washing and other experiments. Three-pole screen-printed electrodes (SPEs) were purchased from Zensor (Taipei, Taiwan) (model TE-100).

### 2.2. Instrumentation

The morphology of the nanoparticles was observed using transmission electron microscopy (TEM) (TEM/JEM 2100 F; JEOL Ltd., Tokyo, Japan). The elemental weight percentages of the resultant materials were analyzed using energy dispersive X-ray analysis (EDX). The crystalline structure was analyzed by X-ray powder diffraction (XRD, X’Pert3 Powder, PANalytical, Almelo, The Netherlands). The chemical bonding of the composites was analyzed by X-ray photoelectron spectroscopy (XPS). Electrochemical experiments were performed using a CH Instruments 6041E workstation (Austin, TX, USA). For the cyclic voltammetry (CV) experiments, the SPE was used as a working, reference, and counter electrode. Amperometry (i-t) experiments were carried out at an optimum potential of 0.49 V using a rotating disc electrode (RDE) as the working electrode, Ag/AgCl as the reference electrode, and a Pt wire as the counter electrode.

### 2.3. Synthesis of PtNi Alloy Nanoparticles

PtNi alloy NPs were synthesized via a facile hydrothermal route using dimethylformamide (DMF) as the solvent. In this process, the nanoparticles were synthesized by dissolving the precursors Pt(acac)_2_ (100 mg) and Ni(NO_3_)_2_.6H_2_O (100 mg) in 80 ml of DMF, and the mixture was stirred continuously with a magnetic stirrer for 30 min. Next, 0.5 g of ascorbic acid (AA) as a reducing agent and 0.8 g of PVP as a surfactant were added to the above mixture, which was stirred for another 30 min. This mixture was hydrothermally treated at 200 °C for 24 h. Next, the PtNi alloy nanoparticles were separated by centrifugation, washed multiple times with hexane and ethanol, and dried in an oven at 50 °C for 12 h.

### 2.4. Fabrication of PtNi-Modified Electrode

The synthesized PtNi nanoparticles (1 mg mL^−1^) were dispersed in DD water via ultrasonic treatment for 20 min to obtain a uniform suspension. The optimized amount (6.0 μL) of the PtNi suspension was coated on the SPE using a micropipette and then dried at 50 °C for 30 min to obtain PtNi/SPE. Bare SPE was used in control studies.

### 2.5. Cell Culture

Breast cancer cells (MDA-MB-231, ATCC, Manassas, VA, USA) and mouse fibroblasts (L929, ATCC, Manassas, VA, USA) were cultured in DMEM supplemented with 10% fetal bovine serum (FBS) (Gibco, Amarillo, TX, USA), 100 U mL^−1^ penicillin, and 100 µg mL^−1^ streptomycin. Cells were seeded in T75 flasks in a CO_2_ incubator maintained at 37 °C, 5% CO_2_, and 95% humidity.

### 2.6. Biocompatibility Analysis

The in vitro cytotoxicity was assessed using the MTT assay. MTT reagent (5 mg mL^−1^) was prepared and stored at 4 °C in the dark. First, the stock solution was diluted to 10-fold using a culture medium. MDA-MB-231 and L929 cells (1 × 10^4^ per well) were cultured in 96 well plates for 24 h to evaluate the in vitro cytotoxicity of the PtNi nanoparticles. After 24 h of incubation, the cells were washed twice with PBS. Next, 50 µL of MTT reagent in 0.5 mL of culture medium was added to the cells and incubated for 4 h in the dark. MTT reagent was then removed from the culture medium, and formazan crystals were dissolved by adding 200 µL of DMSO per well. The plates were shaken using a rotary shaker at 100 rpm for 10 min at room temperature.

### 2.7. Detection of H_2_S in Live Cells

MDA-MB-231 and L929 cells were cultured in T75 flasks for 24 h and grown in DMEM. The cell culture medium was removed after 90% confluence of cells; the cells were then washed thrice with PB and collected by centrifugation. The number of cells was counted using a hemocytometer. Cells (2 × 10^7^) were dispersed in 80 mL of PB (pH 7.0), and vascular endothelial growth factor (VEGF) was used as a stimulator to stimulate H_2_S production. Then, amperometry experiments in live cells were performed at a fixed potential of 0.49 V and VEGF was injected into the PB without cells as a control sample.

## 3. Results

**Figure 1:**
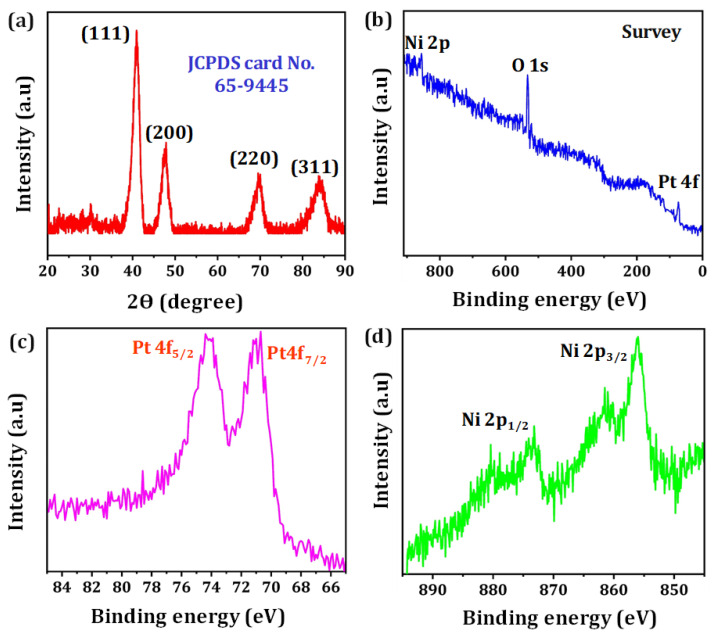
(**a**) XRD pattern of PtNi NPs, (**b**) survey spectrum of PtNi NPs, magnified XPS profiles of (**c**) Pt 4f and (**d**) Ni 2p.

X-ray diffraction (XRD) was employed to examine the crystalline structure of PtNi NPs (Figure 1a). The PtNi shows peaks at 40.2°, 48.2°, 68.3°, and 83.7° corresponding to the (111), (200), (220), and (311) planes, respectively, indicating a face-centered cubic (fcc) structure (JCPDS 65-9445). This indicates the formation of a pure PtNi alloy without any other impurities such as Ni oxide and Pt oxide [19]. In addition, all the diffraction peaks of PtNi NPs were broadened compared to those for Pt, indicating the small size of the nanocrystals of PtNi alloy because Ni diffused into the crystal lattice of Pt, leading to lattice compression.

XPS was employed to study the elemental states and composition of the PtNi NPs. The overall survey of the PtNi alloys in Figure 1b confirms the presence of the individual elements Pt and Ni. Additionally, an intense peak is observed at approximately 530 eV in the survey spectrum due to the presence of atmospheric oxygen atoms. Figure 1c shows a magnified spectrum of Pt 4f, with two peaks located at 74.3 eV and 70.6 eV indexed to Pt 4f_5/2_ and Pt 4f_7/2_ of the PtNi nanoalloy, indicating Pt in the metallic Pt^0^ state. Figure 1d shows two peaks at 852.7 eV and 870.6 eV ascribed to Ni 2p_3/2_ and Ni 2p_1/2_, respectively, indicating the metallic Ni^0^ state [21]. These XPS results further confirm the formation of an alloy nanostructure.

**Figure 2:**
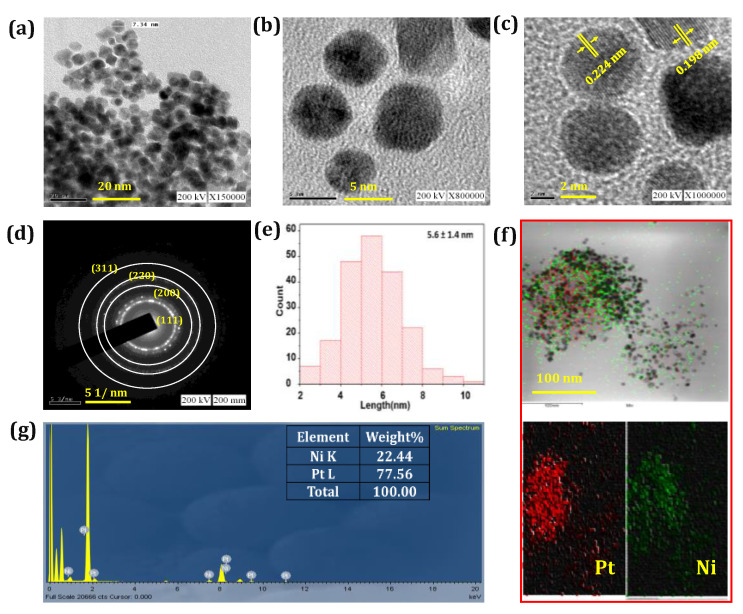
(**a**) TEM image, (**b**,**c**) HRTEM images, (**d**) selected area electron diffraction (SAED) pattern, (**e**) particle size distribution, (**f**) EDX mapping images, (**g**) EDX spectrum of PtNi NPs (insert: elemental weight percentage).

The morphology and nanoparticle size of the PtNi alloy were examined via TEM measurements. The TEM image of the PtNi alloy (Figure 2a) shows identical spherical PtNi alloy NPs with an average diameter of ∼5–6 nm, and Image J software was used to measure their size. Representative high-resolution TEM (HRTEM) images of the PtNi alloy are presented in Figure 2b,c, which exhibit clear and evenly spaced lattice fringes. The lattice spacing distances for the PtNi alloy are 0.198 nm and 0.224 nm, which agrees with the (200) and (111) planes of the PtNi alloys. Figure 2d displays SAED patterns of PtNi nano alloys, which show clear circles ascribed to the (111), (200), (220), and (311) planes, consistent with the XRD results [22]. To further verify the size of the PtNi alloy particles, particle size distribution analysis was performed (Figure 1e), and the average particle size of PtNi NPs was calculated to be 5.6 ± 1.4 nm. The EDS mapping results of the PtNi NPs (Figure 2f) reveal that the Pt and Ni are evenly distributed throughout the particle. Moreover, the weight percentages of Pt and Ni were analyzed by EDX (Figure 2g) and found to be 77.56% and 22.44%, respectively.

### 3.1. Electrochemical Detection of H_2_S at PtNi/SPE

**Figure 3:**
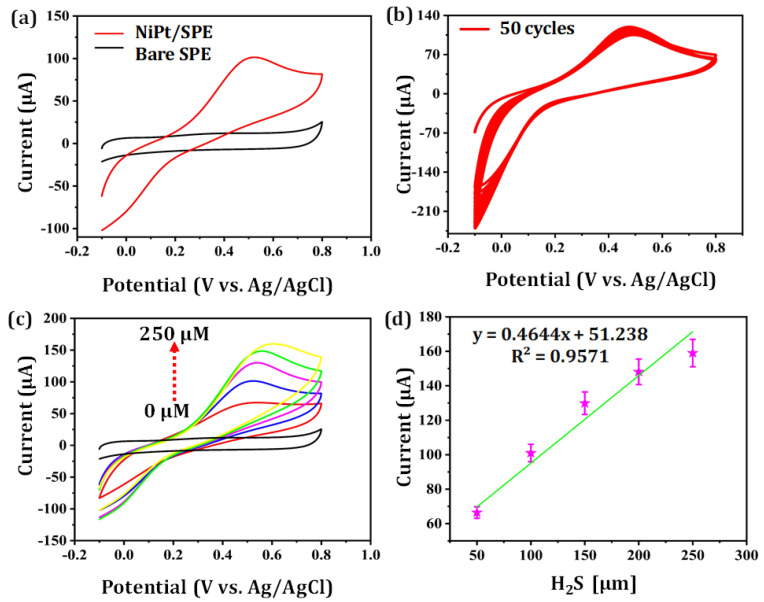
(**a**) CV curves of bare SPE and PtNi/SPE for 100 µM sulfide in 0.05 M PB (pH 7.0) at a scan rate of 0.05 V s^−1^, (**b**) 50 consecutive CV cycles of PtNi/SPE in 0.05 M PB (pH 7.0) containing 100 µM of sulfide at a scan rate of 0.05 V s^−1^, (**c**) CV curves of different concentrations of sulfide (0–250 µM) at PtNi/SPE in 0.05 M PB (pH 7.0), (**d**) calibration plot of I_pa_ vs. concentration (µM) of sulfide.

The electrocatalytic activity of bare SPE and PtNi/SPE towards the oxidation of 100 µM of sulfide was estimated by CV in 0.05 M PB (pH 7.0) at a scan rate of 0.05 V s^−1^ (Figure 3a). In the aqueous solution Na_2_S dissociated into Na^+^, OH^−^, and H_2_S. The bare SPE does not display any noticeable peak for H_2_S, indicating the absence of catalytic sites on the bare SPE surface. On the other hand, SPEs modified with PtNi enhance the peak intensities due to the oxidation of hydrogen sulfide (H_2_S) into bisulfite ion (HS^−^) with the transfer of 2e^−^ and 2H^+^. The higher specific surface area of the PtNi alloy forms a network structure on the surface of the electrode, which is beneficial to increase the catalytic performance of the sensing materials. The stability of PtNi/SPE for the detection of 100 µM sulfide was investigated by repeatedly scanning the PtNi/SPE (50 cycles) (Figure 3b). After 50 cycles, the current response of PtNi/SPE towards sulfide was 85% of its initial (1st cycle) response, indicating outstanding electrochemical stability of the PtNi-modified electrode.

The electrochemical sensitivity of PtNi/SPE was studied at various sulfide concentrations. Figure 3c presents CV curves of sulfide at different concentrations (0–250 µM) on PtNi/SPE in PB (pH 7.0). As the concentration of sulfide increases from 0 to 250 µM, the peak intensity of the oxidation peak of sulfide also gradually increases linearly. The obtained I_pa_ versus sulfide concentration plot is displayed in Figure 3d. The linear regression and coefficient are as follows: I_pa_ (µA) = 0.464 [H_2_S] (µM) + 51.238 and R^2^ = 0.957, respectively.

**Figure 4:**
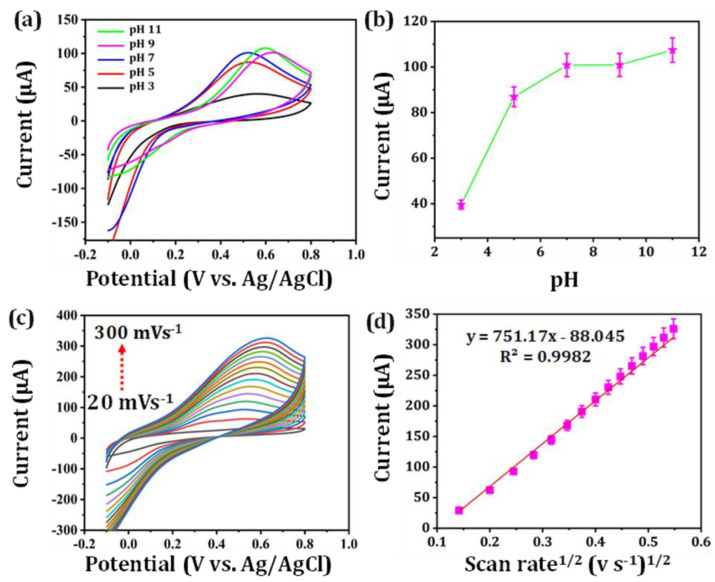
(**a**) CV curves of 100 µM sulfide at the PtNi/SPE in 0.05 M PB at pHs from 3.0 to 11.0 at a scan rate of 0.05 V s^−1^, (**b**) plot of pH vs. current response, (**c**) CV response of PtNi/SPE for 100 µM of sulfide at various scan rates (0.02–0.3 V s^−1^), (**d**) linear plot of I_pa_ of sulfide vs. square root of scan rate (Vs^−1^)^1/2^.

Furthermore, CV was employed to investigate the influence of pH with 100 µM sulfide at PtNi/SPE (Figure 4a). It can be seen that the pH of the 0.05 M PB significantly influences the H_2_S oxidation at the PtNi-modified electrode. Figure 4b shows a plot of the oxidation current of sulfide at PtNi/SPE vs. pH (3.0–11.0). An enhancement in the I_pa_ value was observed from pH 5.0 to 11.0, and the highest I_pa_ was obtained at pH 7.0. Therefore, H_2_S detection is best performed at a pH of 7.0. H_2_S is a weak acid with values of pK_a1_ and pK_a2_, as indicated in Equations (1) and (2):

H_2_S ⇌ HS^−^     pK_a1_ = 6.9 (1)

HS ⇌ S^2−^      pK_a2_ = 14.15 (2)

At a neutral pH of 7.0, sulfide is present as 20% H_2_S, 80% HS^−^, and 0% S^2−^, whereas H_2_S and S^2−^ are the prominent forms at strong acidic and basic pHs, respectively [23]. Of the three forms of sulfide, the most electrochemically detectable form is HS^−^. Moreover, the H_2_S oxidation rate in strong acid or strong base is sluggish because of the lower availability of the HS^−^ form. Neutral PB with a pH of 7.0 is essential to create a suitable environment for the conversion of H_2_S to HS^−^; therefore, pH 7.0 was selected for further studies.

The electrokinetic behavior of PtNi/SPE was studied by CV at various scan rates (20–300 mVs^−1^) for 100 µM sulfide (Figure 4c). When the scan rate increases from 0.02 to 0.3 V s^−1^, the I_pa_ of sulfide also increases linearly and the oxidation peak potential (E_pa_) is shifted towards the positive direction. A linear plot was obtained between the I_pa_ of sulfide and the square root of the scan rate, with a linear regression equation I_pa_ (µA) = 751.17 v^1/2^ (Vs^−1^)^1/2^ − 88.045 and a regression coefficient R^2^ = 0.9933 (Figure 4d), indicating that the electrochemical oxidation of sulfide on PtNi/SPE is a diffusion-controlled process.

### 3.2. Amperometric Determination of H_2_S at PtNi/SPE

**Figure 5:**
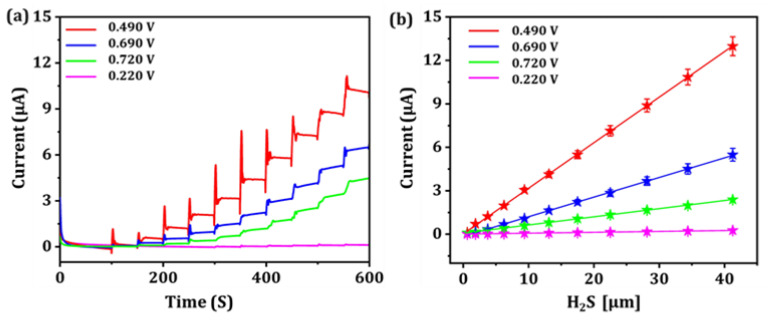
(**a**) Amperometry (i-t) response of the PtNi/SPE with different concentrations of sulfide (0.625–41.2 µM) in 0.05 M PB (pH 7.0) at various working potential ranges (0.49, 0.69, 0.72, and 0.22 V), (**b**) corresponding linear plot of I_pa_ vs. [H_2_S] for different potentials.

Before determining the typical amperometric response for H_2_S, it is necessary to determine the optimum applied potential for efficient H_2_S detection. Current-time (i-t) curves were recorded at different potentials (0.49, 0.69, 0.72, and 0.22 V vs. Ag/AgCl) with successive additions of sulfide (0.625–41.2 µM). A higher and more stable I_pa_ with lower background noise was obtained at 0.49 V compared to that at other potentials (Figure 5a). A linear relationship was observed between I_pa_ and [H_2_S] over a wide range of potentials (Figure 5b). Therefore, a potential of 0.49 V was selected as the most suitable potential for the subsequent amperometric H_2_S detection measurements.

**Figure 6:**
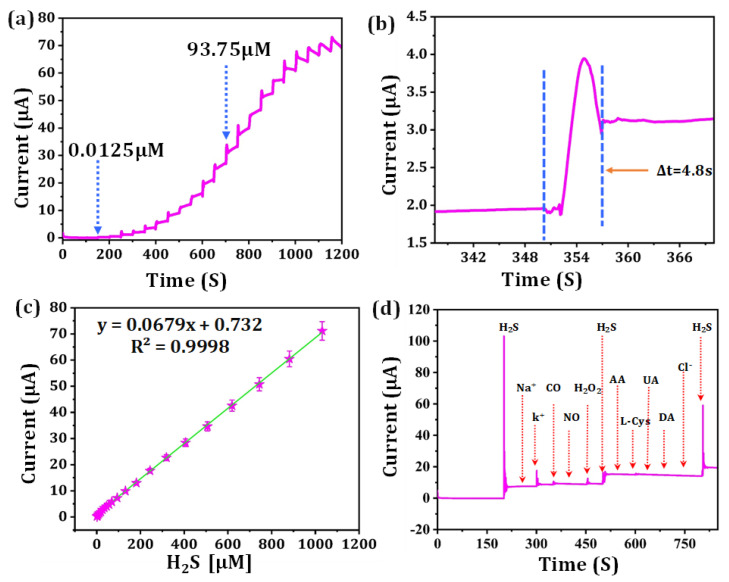
(**a**) Amperometry (i-t) response of various concentrations of sulfide (0.0125–1031 µM) in 0.05 M PB (pH 7.0), (**b**) response time for steady-state current, (**c**) corresponding linear plot for I_pa_ vs. [sulfide] (**d**) effect of interfering species Na^+^, K^+^, CO, NO, hydrogen peroxide (H_2_O_2_), ascorbic acid (AA), L-cysteine (L-cys), uric acid (UA), dopamine (DA), and Cl^−^ on the amperometric responses of PtNi/SPE for sulfide in 0.05 M PB (pH 7.0).

After determining the optimum potential, the sensing performance of PtNi towards sulfide was investigated. Figure 6a illustrates the amperometric (i-t) response of PtNi with successive additions of various concentrations of sulfide into 0.05 M PB (pH 7.0) at regular intervals of 50 s under stirring. As depicted in Figure 6a, the I_pa_ increased quickly with the successive additions of sulfide, and reached a steady-state current within 4.8 s after each addition (Figure 6b). This result demonstrates electrochemical activity and quick adsorption of H_2_S molecules on the PtNi electrode surface. The corresponding amperometric current response versus sulfide concentration is shown in Figure 6c. A linear relationship was found for PtNi in the range 0.0125–1031 µM with a linear regression equation I_pa_ (µA) = 0.0679 [H_2_S] (µM) + 0.732 and a coefficient R^2^ = 0.999. Based on the limit of detection (LOD) = 3S/k, the LOD was determined to be 0.004 µM, where ‘k’ indicates the slope value (0.0679 µA µM^−1^) achieved from the linear plot, and ‘S’ indicates the standard deviation from three blank measurements (0.0001 µA). Moreover, the sensitivity of the PtNi sensor was calculated to be 0.323 μA μM^−1^ cm^−2^. The obtained linear range and LOD of our PtNi sensor were compared with those of previously reported H_2_S sensors, as displayed in Table 1. In comparison, the analytical performance of the proposed PtNi sensor was superior to that of most of the other proposed H_2_S detection methods.

An interference study was performed to investigate the selectivity of PtNi toward the oxidation of sulfide with other interfering compounds. The current response for the consecutive addition of sulfide and some potential interferences were investigated in PB (pH 7.0) at 0.49 V on a PtNi sensor (Figure 6d). A 10-fold excess concentration of Na^+^, K^+^, CO, NO, hydrogen peroxide (H_2_O_2_), uric acid (UA), ascorbic acid (AA), L-cysteine (L-cys), dopamine (DA), and Cl^−^ had no substantial influence on the amperometric response; however, the addition of sulfide resulted in a notable change in the amperometric response. These results suggest that the PtNi sensor has excellent selectivity towards H_2_S oxidation and exhibits high anti-fouling by the interfering compounds.

### 3.3. Repeatability and Reproducibility

**Figure 7:**
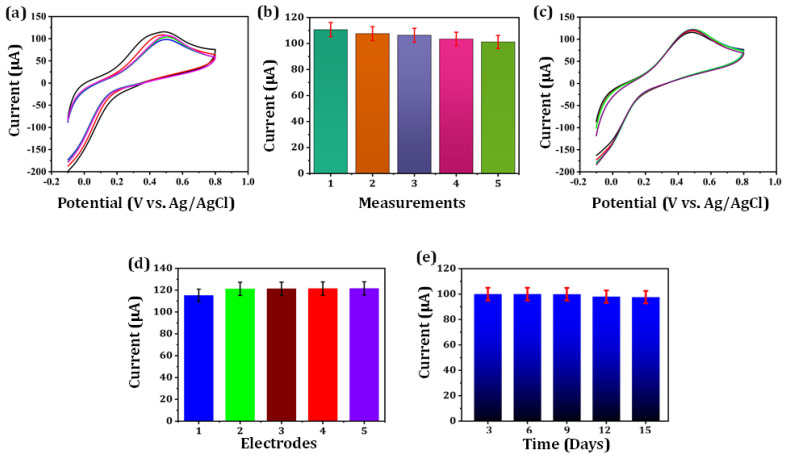
(**a**) Repeatability of the PtNi/SPE for 5 independent measurements in 0.05 M PB (pH-7.0) containing 100 µM sulfide, (**b**) bar diagram of I_pa_ and number of measurements, (**c**) Reproducibility of the PtNi/SPE, (**d**) bar diagram of I_pa_ and number of PtNi/SPE, (**e**) stability bar diagram of PtNi/SPE over 15 days storage.

The repeatability and reproducibility of the PtNi-modified electrodes were investigated by CV in 0.05 M PB (pH 7.0) containing 100 μM sulfide. The repeatability of the single PtNi/SPE toward sulfide was studied by observing the current response over five independent CV measurements under the same experimental conditions (Figure 7a). A relative standard deviation (RSD) of 3.37% was estimated from the bar diagram of I_pa_ and the number of measurements (Figure 7b). A series of five different PtNi/SPE electrodes were prepared to evaluate the reproducibility of the PtNi/SPE, and the I_pa_ of 100 µM sulfide on each electrode was observed by CV (Figure 7c). As shown in the bar diagram (Figure 7d), small changes in the I_pa_ value with an RSD of 3.89% demonstrated the excellent reproducibility of the PtNi/SPE sensor. In order to investigate the long-term stability of the proposed sensor, the current response of the PtNi electrode was analyzed by 100 µM of the H_2_S at the single electrode for 15 days. The current responses were measured every 3 days intervals after the PtNi electrode was stored in the refrigerator at 4°C (Figure 7e). The PtNi electrode retained 93.7% of current from its first day and the RSD value was calculated to be 4.82%; the obtained result revealed the excellent long-term stability of our proposed sensor.

### 3.4. Real Sample Analysis

**Figure 8:**
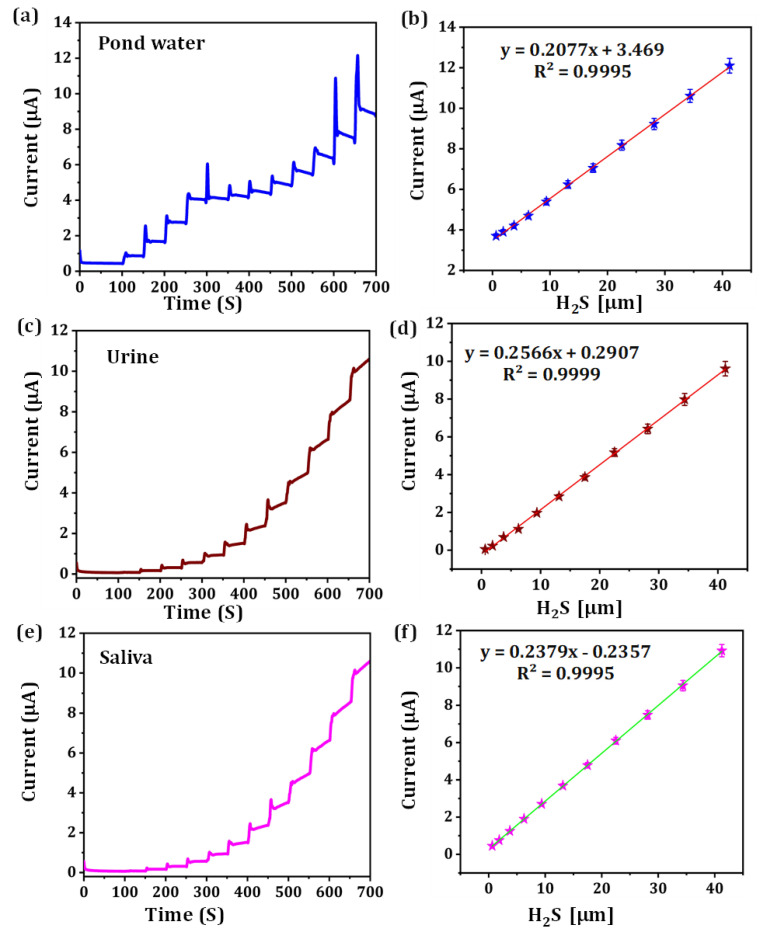
Detection of H_2_S at PtNi-modified electrode in (**a**) pond water, (**c**) urine, and (**e**) saliva samples containing 0.05 M PB (pH 7.0), with corresponding plots of I_pa_ vs. [H_2_S] for (**b**) pond water, (**d**) urine, and (**f**) saliva samples.

The practicality of the PtNi sensor was scrutinized by amperometric (i-t) measurements of sulfide in pond water, human urine, and saliva samples. The amperometric (i-t) response was recorded at 0.49 V in 0.05 M PB (pH 7.0) with consecutive additions of real samples containing sulfide (0.625–41.25 µM). A stock solution of pond water was diluted 10 times with PB, a known concentration of Na_2_S was injected into the stock solution, and the amperometric (i-t) response was observed for different consecutive additions of stock solution (0.625–41.25 µM) in 0.05 M PB (pH 7.0) (Figure 8a). Similarly, collected human urine and saliva samples were separately diluted with 0.05 M PB, and Whatman filter paper was used to filter them. Subsequently, a known concentration of sulfide was added to these real samples, and the amperometric (i-t) response was recorded using the standard addition method, as depicted in Figure 8c,e, respectively. Linear plots of I_pa_ vs. [sulfide] for pond water, human urine, and human saliva samples are displayed in Figure 8b,d,f. The recovery results are listed in Table 2. The PtNi sensor exhibited good recovery values for all three real samples, with average recoveries of 92.4%, 95.6%, and 98.8% for pond water, human urine, and human saliva samples, respectively. These results suggest that the PtNi sensor can be utilized as a reliable platform for the quantification of H_2_S in real samples.

### 3.5. In Vitro Biological Sample Analysis

**Figure 9:**
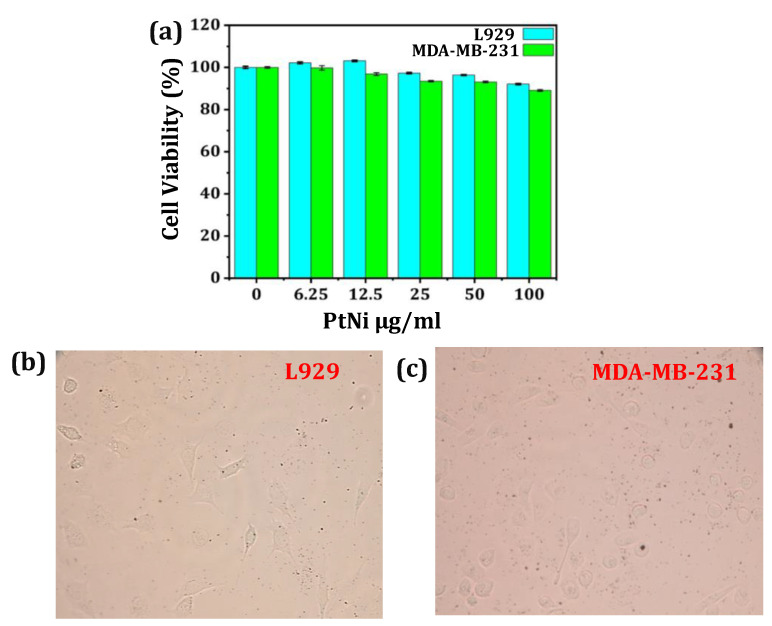
(**a**) Cell viability of L929 and MDA-MB-231 cells, microscopic images of (**b**) L929 and (**c**) MDA-MB-231 cells.

Before the electrochemical detection of H_2_S in live cells, the biocompatibility of PtNi was investigated. L929 and MDA-MB-231 cells were cultured with different concentrations of PtNi (6.25, 12.5, 25, 50, and 100 µg ml^−1^) and an MTT assay was performed to evaluate cell viability. Cell survival was inhibited in a concentration-dependent manner. The L929 and MDA-MB-231 cells showed more than 90% viability at 100 µg ml^−1^ (Figure 9a), indicating good biocompatibility of the as-prepared material. Therefore, PtNi could be useful for in vitro and in vivo analyses. Figure 9b,c shows the microscopic images of L929 and MDA-MB-231 cells cultured with PtNi alloy nanoparticles. The cells were healthy and proliferated well after 24 h of incubation with PtNi (10 µg ml^−1^).

**Figure 10:**
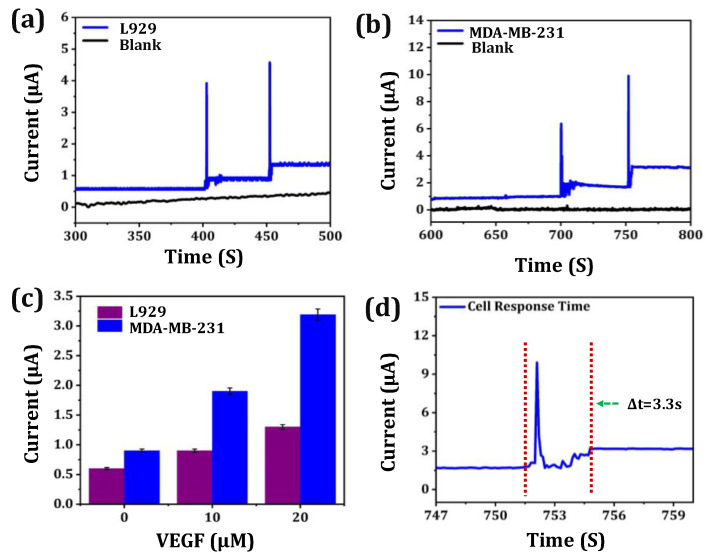
(**a**) Amperometric responses of PtNi to consecutive additions of VEGF (0, 10, and 20 µM) without L929 and with L929 cells, (**b**) amperometric responses of PtNi-modified electrode to consecutive additions of VEGF stimulator (0, 10, and 20 µM) without MDA-MB-231 and with MDA-MB-231 cells in 0.05 M PB at 0.49 V, (**c**) corresponding current response between L929 and MDA-MB-231 cells with various concentrations of VEGF, (**d**) amperometric response time for H_2_S released from cancer cells.

Amperometric analysis was performed to detect H_2_S released from L929 and MDA-MB-231 cells. VEGF was used as a stimulator for H_2_S release from live cells. The L929 and MDA-MB-231 cells were cultured separately and tested using the amperometric technique in 0.05 M PB at the optimum potential of 0.49 V. A control experiment was performed in PB (7.0) without L929 and MDA-MB-231 cells. The current response did not change in these cells when VEGF was added to the PB. However, the current response increased with consecutive additions of VEGF (0, 10, and 20 µM) into PB containing L929 cells and MDA-MB-231, as shown in Figure 10a,b, respectively. The corresponding current response of H_2_S released from L929 and MDA-MB-231 cells is shown in Figure 10c. The current response from MDA-MB-231 cells was 3.4-fold higher than that from L929 cells, confirming that cancer cells produce a higher concentration of H_2_S than normal cells. These results suggest that PtNi could be a potential candidate for monitoring H_2_S in live cells. The amperometry detection time for H_2_S released from cancer cells was presented in Figure 10d. As depicted in Figure 10d, the I_pa_ increased quickly with the successive additions of VEGF into PB-containing cells and reached a steady-state current within 3.3 s after the addition of VEGF. This result demonstrates the quick adsorption of H_2_S molecules on the PtNi electrode surface.

## 4. Conclusions

Herein, PtNi alloy nanoparticles were successfully prepared using a hydrothermal method for the detection of H_2_S. The average diameter of ∼5–6 nm of PtNi NPs were scrutinized by TEM analysis. Additionally, EDX, XRD, and XPS observations confirmed the successful formation of PtNi alloys. The PtNi sensor showed outstanding catalytic performance toward hydrogen sulfide oxidation with an LOD of 0.004 μM, sensitivity of 0.323 μA μM^−1^ cm^−2^, and a linear detection range of 0.013–1031 μM. The PtNi sensor shows excellent selectivity towards H_2_S oxidation and exhibits high anti-fouling by the interfering compounds. The repeatability, reproducibility, and long-term stability of the PtNi sensor were successfully demonstrated with RSD value of 3.37%, 3.89%, and 4.82%, respectively. The practical applicability of the PtNi sensor based on the quantification of H_2_S in real samples such as pond water, human urine, and saliva samples was demonstrated with average recoveries of 92.4%, 95.6%, and 98.8%, respectively. Finally, we demonstrated that the PtNi sensor could be effectively applied for the detection of H_2_S in live cells. Our results indicated that cancer cells (MDA-MB-231) released 3.4-fold higher levels of H_2_S than normal cells (L929). Hence, the reported method has high potential for biomedical research related to H_2_S production.

## Figures and Tables

**Table 1:** Comparison of sensor parameters (linear range and LOD) for the proposed PtNi sensor with previously reported H_2_S sensors.

Modified Electrodes	Type	Linear Range (µM)	LOD (µM)	Ref.
Cu_2_O−CuO@AuNIs ODH	Amperometry	1–11,780	0.7	[13]
magnetic CNT/Co/MoS_2_	Amperometry	0.25–13,327	0.12	[24]
Escherichia coli/NPG	Cyclic voltammetry	0.4–33,000	0.026	[25]
Ti_3_C_2_Tx MXene	Amperometry	0.1–300	0.016	[26]
Boron Doped Diamond Flow Electrodes	Flow injection Amperometry	0−51.2	0.37 ± 0.03	[10]
Electropolymerized film-modified GCEs	Amperometry	0–15	0.1	[1]
PtNi	Amperometry	0.0125–1031	0.004	Present work

**Table 2:** Detection of H_2_S in pond water, urine, and saliva samples with the PtNi-modified electrode.

Samples	Added (µM)	Found (µM)	Recovery (%)	* RSD (%)
Pond water	0	0	-	3.39
	10	9.24	92.4	
Human urine	0	0	-	4.2
	10	9.56	95.6	
Saliva	0	0	-	4.65
	10	9.88	98.8	

* Related standard deviation (RSD) of three independent experiments.

## Data Availability

Not applicable.
